# 2-*O*-Benzhydryl-3,4-(*S*)-*O*-benzyl­idene-d-xylono-1,4-lactone

**DOI:** 10.1107/S1600536808012567

**Published:** 2008-05-07

**Authors:** Sarah F. Jenkinson, Sebastian D. Rule, Kathrine V. Booth, George W. J. Fleet, David J. Watkin, Sigthor Petursson

**Affiliations:** a Department of Organic Chemistry, Chemical Research Laboratory, University of Oxford, Mansfield Road, Oxford OX1 3TA, England; b Department of Chemical Crystallography, Chemical Research Laboratory, University of Oxford, Mansfield Road, Oxford OX1 3TA, England; c Faculty of Business and Science, University of Akureyri, IS-600 Akureyri, Iceland

## Abstract

X-ray crystallography unequivocally shows that protection of the free hydroxyl group of 3,5-*O*-benzyl­idene-d-xylono-1,4-lactone with diphenyl­diazo­methane proceeded smoothly to give the title compound, C_25_H_22_O_5_, with no accompanying epimerization. Unlike the analogously protected *lyxono* lactone, the isomeric *xylono* lactone has two mol­ecules present in the asymmetric unit (*Z*′ = 2). The 5-ring lactones adopt envelope conformations and the 6-ring ketals adopt chair conformations.

## Related literature

For related literature, see: Collins & Ferrier (1995 ▶); Draths *et al.* (1992 ▶); Jackson *et al.* (1982 ▶); Petursson & Webber (1982 ▶); Petursson *et al.* (2007 ▶); Petursson (2001 ▶, 2003 ▶); Best *et al.* (2008 ▶); Jenkinson *et al.* (2008 ▶); Görbitz (1999 ▶).
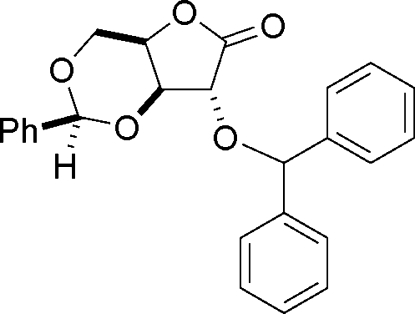


## Experimental

### 

#### Crystal data

C_25_H_22_O_5_
*M* *_r_* = 402.45
Monoclinic, 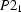

*a* = 14.8159 (3) Å
*b* = 9.1959 (2) Å
*c* = 15.0797 (2) Å
β = 93.7245 (12)°
*V* = 2050.20 (7) Å^3^
*Z* = 4
Mo *K*α radiation
μ = 0.09 mm^−1^
*T* = 150 K
0.80 × 0.30 × 0.10 mm

#### Data collection

Nonius KappaCCD area-detector diffractometer
Absorption correction: multi-scan (*DENZO*/*SCALEPACK*; Otwinowski & Minor, 1997 ▶) *T*_min_ = 0.55, *T*_max_ = 0.99
25081 measured reflections
4938 independent reflections
3950 reflections with *I* > 2σ(*I*)
*R*_int_ = 0.047

#### Refinement

*R*[*F* ^2^ > 2σ(*F* ^2^)] = 0.034
*wR*(*F* ^2^) = 0.101
*S* = 0.89
4938 reflections
542 parameters
1 restraint
H-atom parameters constrained
Δρ_max_ = 0.25 e Å^−3^
Δρ_min_ = −0.27 e Å^−3^

### 

Data collection: *COLLECT* (Nonius, 2001 ▶).; cell refinement: *DENZO*/*SCALEPACK* (Otwinowski & Minor, 1997 ▶); data reduction: *DENZO*/*SCALEPACK*; program(s) used to solve structure: *SIR92* (Altomare *et al.*, 1994 ▶); program(s) used to refine structure: *CRYSTALS* (Betteridge *et al.*, 2003 ▶); molecular graphics: *CAMERON* (Watkin *et al.*, 1996 ▶); software used to prepare material for publication: *CRYSTALS*.

## Supplementary Material

Crystal structure: contains datablocks global, I. DOI: 10.1107/S1600536808012567/lh2623sup1.cif

Structure factors: contains datablocks I. DOI: 10.1107/S1600536808012567/lh2623Isup2.hkl

Additional supplementary materials:  crystallographic information; 3D view; checkCIF report

## Figures and Tables

**Fig. 1.**
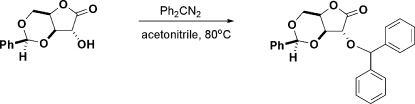
Synthetic Scheme.

**Fig. 2.**
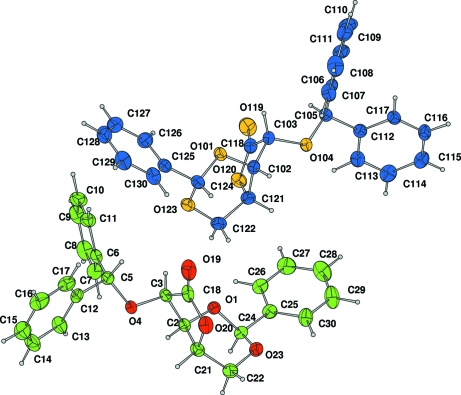
The title compound with displacement ellipsoids drawn at the 50% probability level. H atoms are shown as spheres of arbitary radius. There are two molecules in the asymmetric repeating unit.

**Fig. 3.**
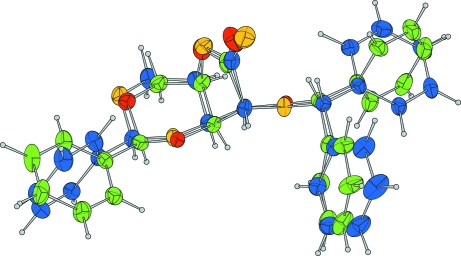
Overlay of the two molecules in the asymmetric unit.

**Fig. 4.**
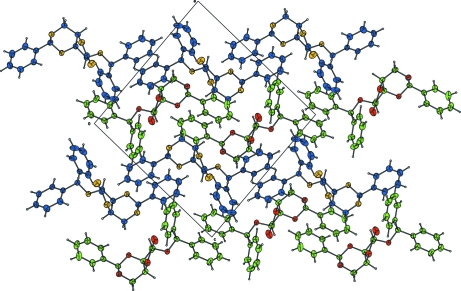
Packing diagram for the molecule projected along the b-axis.

## supplementary crystallographic information

### Comment

Carbohydrates provide excellent starting materials for the synthesis of small chiral molecules (Collins & Ferrier, 1995). They are relatively inexpensive and provide an almost boundless pool of chiral building blocks (Draths *et al.*, 1992). Much of their synthetic utility is however dependent on developing successful protecting group strategies.

Diazodiphenylmethane has been found to be a useful protecting group in the synthesis of methyl 2,3,6-tri-*O*-methyl-α-*D*-glucopyranoside and kojibiose octa-acetate (Jackson *et al.*, 1982); monoalkylations of vicinal diols have been achieved with this reagent and other diaryldiazoalkanes with high regioselectivities (Petursson & Webber, 1982; Petursson *et al.*, 2007; Petursson, 2003; Petursson, 2001). This is of particular interest for the protection of base sensitive sugar lactones as the reaction is carried out under neutral conditions (Best *et al.* 2008; Jenkinson *et al.* 2008).

The utility of the benzhydryl group as a protecting group in carbohydrate chemistry has here been demonstrated with the reaction of 3,5-*O*-benzylidene-D-xylono-1,4-lactone 1 with diphenyldiazomethane (Fig. 1). No epimerization at C2 was observed (Fig. 2).

Unlike the analogously protected *lyxono* lactone (Jenkinson *et al.*, 2008), the asymmetric unit of the isomeric *xylono* lactone contains two crystallographically distinct molecules which are related by a pseudo 2-fold axis of symmetry. These are similar in geometry with the exception of two of the phenyl rings which sit at approximately 90° to each other (Fig. 3). When the core 20 atoms, of the carbohydrate backbone and 1 phenyl group, are mapped there is good overlap- r.m.s. deviations: posn 0.1139 Å, bond 0.0104 Å, torsion 2.5205°.

The crystal packing shows alternating layers of molecules in the *ac* plane (Fig. 4). The 5-ring lactones adopt envelope conformations with C2 or C102 out of the plane. The 6-ring ketals adopt chair conformations. There is no classic hydrogen-bonding.

### Experimental

The title lactone was recrystallized from a 1:1 mixture of ethyl acetate and cyclohexane: m.p. 395–397 K; [α]_D_^23^ +129.1 (*c*, 1.02 in CHCl_3_).

### Refinement

In the absence of significant anomalous scattering, Friedel pairs were merged and the absolute configuration was assigned from the starting material.

The relatively large ratio of minimum to maximum corrections applied in the multiscan process (1:1.80) reflect changes in the illuminated volume of the crystal. Changes in illuminated volume were kept to a minimum, and were taken into account (Görbitz, 1999) by the multi-scan inter-frame scaling (*DENZO*/*SCALEPACK*, Otwinowski & Minor, 1997).

The H atoms were all located in a difference map, but those attached to carbon atoms were repositioned geometrically. The H atoms were initially refined with soft restraints on the bond lengths and angles to regularize their geometry (C—H in the range 0.93–0.98, O—H = 0.82 Å) and *U*_iso_(H) (in the range 1.2–1.5 times *U*_eq_ of the parent atom), after which the positions were refined with riding constraints.

### Crystal data

**Table:**

C_25_H_22_O_5_	*F*_000_ = 848
*M**_r_* = 402.45	*D*_x_ = 1.304 Mg m^−^^3^
Monoclinic, *P*2_1_	Mo *K*α radiation λ = 0.71073 Å
Hall symbol: P 2yb	Cell parameters from 4834 reflections
*a* = 14.8159 (3) Å	θ = 5–27º
*b* = 9.1959 (2) Å	µ = 0.09 mm^−^^1^
*c* = 15.0797 (2) Å	*T* = 150 K
β = 93.7245 (12)º	Plate, colourless
*V* = 2050.20 (7) Å^3^	0.80 × 0.30 × 0.10 mm
*Z* = 4	

### Data collection

**Table:**

Nonius KappaCCD area-detector diffractometer	3950 reflections with *I* > 2σ(*I*)
Monochromator: graphite	*R*_int_ = 0.047
*T* = 150 K	θ_max_ = 27.5º
ω scans	θ_min_ = 5.2º
Absorption correction: multi-scan(DENZO/SCALEPACK; Otwinowski & Minor, 1997)	*h* = −19→19
*T*_min_ = 0.55, *T*_max_ = 0.99	*k* = −11→11
25081 measured reflections	*l* = −19→19
4938 independent reflections	

### Refinement

**Table:**

Refinement on *F*^2^	Hydrogen site location: inferred from neighbouring sites
Least-squares matrix: full	H-atom parameters constrained
*R*[*F*^2^ > 2σ(*F*^2^)] = 0.034	Method, part 1, Chebychev polynomial, (Watkin, 1994, *P*rince, 1982) [*w*eight] = 1.0/[A_0_*T_0_(x) + A_1_*T_1_(x) ··· + A_n-1_]*T_n-1_(x)] where A_i_ are the Chebychev coefficients listed belo*w* and x = *F* /*F*max Method = Robust Weighting (*P*rince, 1982) W = [*w*eight] * [1-(delta*F*/6*sigma*F*)^2^]^2^ A_i_ are: 16.9 24.9 12.6 3.46
*wR*(*F*^2^) = 0.101	(Δ/σ)_max_ = 0.0003
*S* = 0.89	Δρ_max_ = 0.25 e Å^−^^3^
4938 reflections	Δρ_min_ = −0.27 e Å^−^^3^
542 parameters	Extinction correction: Larson (1970), Equation 22
1 restraint	Extinction coefficient: 600 (50)
Primary atom site location: structure-invariant direct methods	

### Fractional atomic coordinates and isotropic or equivalent isotropic displacement parameters (Å^2^)

**Table:**

	*x*	*y*	*z*	*U*_iso_*/*U*_eq_	
O1	0.44166 (10)	0.5327 (2)	0.31715 (10)	0.0244	
C2	0.40499 (15)	0.5336 (3)	0.22694 (15)	0.0242	
C3	0.30725 (16)	0.4866 (3)	0.23203 (15)	0.0254	
O4	0.25857 (10)	0.5219 (2)	0.15093 (10)	0.0265	
C5	0.16129 (15)	0.5192 (3)	0.15751 (16)	0.0257	
C6	0.13142 (16)	0.6490 (3)	0.21052 (15)	0.0252	
C7	0.17991 (18)	0.7781 (3)	0.21395 (18)	0.0330	
C8	0.1517 (2)	0.8947 (3)	0.26476 (19)	0.0403	
C9	0.0760 (2)	0.8806 (3)	0.31222 (18)	0.0432	
C10	0.0264 (2)	0.7539 (4)	0.30826 (19)	0.0437	
C11	0.05367 (19)	0.6375 (3)	0.25762 (17)	0.0335	
C12	0.12135 (15)	0.5140 (3)	0.06274 (15)	0.0255	
C13	0.15139 (19)	0.6107 (3)	0.00038 (17)	0.0339	
C14	0.1185 (2)	0.6028 (4)	−0.08784 (18)	0.0429	
C15	0.0551 (2)	0.4987 (4)	−0.11333 (19)	0.0486	
C16	0.02472 (19)	0.4030 (4)	−0.0519 (2)	0.0479	
C17	0.05739 (17)	0.4106 (4)	0.03651 (19)	0.0368	
C18	0.31556 (18)	0.3212 (3)	0.23435 (18)	0.0323	
O19	0.26098 (14)	0.2338 (2)	0.25627 (16)	0.0478	
O20	0.39494 (13)	0.2813 (2)	0.20131 (13)	0.0344	
C21	0.44429 (17)	0.4094 (3)	0.17435 (16)	0.0296	
C22	0.54422 (17)	0.3886 (3)	0.19447 (16)	0.0335	
O23	0.56825 (11)	0.3965 (2)	0.28758 (11)	0.0301	
C24	0.53776 (15)	0.5268 (3)	0.32352 (15)	0.0255	
C25	0.56837 (16)	0.5320 (3)	0.42104 (16)	0.0277	
C26	0.53029 (17)	0.6335 (3)	0.47592 (17)	0.0318	
C27	0.5559 (2)	0.6362 (4)	0.56635 (19)	0.0417	
C28	0.6204 (2)	0.5396 (4)	0.6014 (2)	0.0479	
C29	0.6607 (2)	0.4425 (4)	0.5464 (2)	0.0479	
C30	0.63432 (19)	0.4375 (3)	0.45597 (19)	0.0359	
O101	0.17514 (11)	0.3628 (2)	0.56763 (10)	0.0280	
C102	0.26545 (16)	0.3497 (3)	0.60615 (15)	0.0265	
C103	0.26225 (16)	0.3956 (3)	0.70212 (16)	0.0266	
O104	0.34196 (11)	0.3412 (2)	0.74916 (10)	0.0274	
C105	0.33862 (16)	0.3463 (3)	0.84483 (15)	0.0254	
C106	0.27885 (16)	0.2264 (3)	0.87746 (17)	0.0275	
C107	0.25628 (19)	0.1047 (3)	0.82618 (19)	0.0368	
C108	0.2043 (2)	−0.0056 (4)	0.8592 (2)	0.0473	
C109	0.17436 (19)	0.0054 (4)	0.9435 (3)	0.0506	
C110	0.1966 (2)	0.1261 (4)	0.9956 (2)	0.0512	
C111	0.2483 (2)	0.2372 (4)	0.9627 (2)	0.0394	
C112	0.43660 (16)	0.3392 (3)	0.88150 (15)	0.0254	
C113	0.49799 (18)	0.4396 (3)	0.85136 (18)	0.0320	
C114	0.58750 (19)	0.4422 (3)	0.8863 (2)	0.0383	
C115	0.61626 (18)	0.3427 (3)	0.95059 (18)	0.0348	
C116	0.55682 (18)	0.2406 (3)	0.97998 (16)	0.0318	
C117	0.46694 (17)	0.2387 (3)	0.94582 (15)	0.0276	
C118	0.27224 (17)	0.5609 (3)	0.69587 (17)	0.0307	
O119	0.25584 (16)	0.6510 (3)	0.75019 (14)	0.0446	
O120	0.30885 (13)	0.5960 (2)	0.61933 (12)	0.0333	
C121	0.32735 (16)	0.4648 (3)	0.56966 (16)	0.0299	
C122	0.30971 (17)	0.4926 (4)	0.47132 (17)	0.0357	
O123	0.21497 (11)	0.4979 (2)	0.44632 (11)	0.0315	
C124	0.17243 (16)	0.3695 (3)	0.47308 (15)	0.0280	
C125	0.07503 (16)	0.3717 (3)	0.43830 (16)	0.0286	
C126	0.01418 (18)	0.4674 (3)	0.47230 (17)	0.0354	
C127	−0.07582 (19)	0.4661 (4)	0.44074 (19)	0.0426	
C128	−0.10512 (19)	0.3684 (4)	0.3750 (2)	0.0417	
C129	−0.0444 (2)	0.2749 (4)	0.3393 (2)	0.0412	
C130	0.0462 (2)	0.2761 (3)	0.37099 (18)	0.0344	
H21	0.4102	0.6308	0.1980	0.0310*	
H31	0.2815	0.5292	0.2838	0.0300*	
H51	0.1461	0.4285	0.1912	0.0293*	
H71	0.2332	0.7897	0.1793	0.0378*	
H81	0.1854	0.9828	0.2657	0.0498*	
H91	0.0574	0.9586	0.3462	0.0522*	
H101	−0.0270	0.7456	0.3417	0.0499*	
H111	0.0193	0.5507	0.2559	0.0398*	
H131	0.1944	0.6831	0.0155	0.0428*	
H141	0.1417	0.6660	−0.1305	0.0511*	
H151	0.0293	0.4918	−0.1722	0.0598*	
H161	−0.0195	0.3290	−0.0668	0.0570*	
H171	0.0384	0.3446	0.0768	0.0465*	
H211	0.4312	0.4257	0.1074	0.0355*	
H221	0.5780	0.4629	0.1587	0.0427*	
H222	0.5617	0.2896	0.1766	0.0439*	
H241	0.5627	0.6105	0.2909	0.0328*	
H261	0.4846	0.7013	0.4543	0.0393*	
H271	0.5298	0.7065	0.6043	0.0510*	
H281	0.6372	0.5419	0.6640	0.0566*	
H291	0.7087	0.3765	0.5704	0.0512*	
H301	0.6622	0.3686	0.4199	0.0430*	
H1021	0.2909	0.2513	0.5939	0.0322*	
H1031	0.2066	0.3671	0.7308	0.0308*	
H1051	0.3131	0.4449	0.8589	0.0297*	
H1071	0.2755	0.0977	0.7683	0.0447*	
H1081	0.1886	−0.0904	0.8241	0.0582*	
H1091	0.1414	−0.0692	0.9676	0.0609*	
H1101	0.1777	0.1334	1.0531	0.0637*	
H1111	0.2619	0.3211	0.9967	0.0452*	
H1131	0.4790	0.5064	0.8020	0.0373*	
H1141	0.6288	0.5114	0.8631	0.0459*	
H1151	0.6769	0.3437	0.9745	0.0399*	
H1161	0.5788	0.1694	1.0226	0.0391*	
H1171	0.4223	0.1678	0.9652	0.0343*	
H1211	0.3914	0.4381	0.5851	0.0389*	
H1221	0.3378	0.5873	0.4563	0.0426*	
H1222	0.3394	0.4138	0.4383	0.0433*	
H1241	0.2031	0.2834	0.4506	0.0363*	
H1261	0.0340	0.5326	0.5187	0.0406*	
H1271	−0.1184	0.5305	0.4660	0.0513*	
H1281	−0.1677	0.3670	0.3552	0.0470*	
H1291	−0.0649	0.2101	0.2929	0.0471*	
H1301	0.0895	0.2105	0.3457	0.0399*	

### Atomic displacement parameters (Å^2^)

**Table:**

	*U*^11^	*U*^22^	*U*^33^	*U*^12^	*U*^13^	*U*^23^
O1	0.0232 (7)	0.0288 (9)	0.0210 (7)	−0.0002 (7)	0.0005 (6)	−0.0019 (7)
C2	0.0265 (11)	0.0255 (12)	0.0202 (10)	0.0009 (9)	−0.0007 (8)	0.0009 (9)
C3	0.0275 (11)	0.0277 (13)	0.0209 (10)	−0.0006 (10)	−0.0001 (8)	0.0048 (10)
O4	0.0243 (8)	0.0340 (10)	0.0209 (7)	0.0030 (7)	−0.0011 (6)	0.0044 (7)
C5	0.0245 (11)	0.0251 (12)	0.0276 (11)	−0.0001 (10)	0.0017 (8)	0.0027 (10)
C6	0.0300 (11)	0.0240 (11)	0.0211 (10)	0.0031 (10)	−0.0022 (8)	0.0024 (9)
C7	0.0352 (13)	0.0288 (13)	0.0346 (13)	−0.0016 (11)	−0.0004 (10)	−0.0027 (11)
C8	0.0518 (16)	0.0289 (14)	0.0387 (14)	−0.0017 (13)	−0.0081 (12)	−0.0037 (12)
C9	0.0659 (19)	0.0334 (16)	0.0298 (13)	0.0140 (15)	0.0001 (12)	−0.0040 (12)
C10	0.0570 (18)	0.0398 (17)	0.0361 (15)	0.0146 (14)	0.0169 (13)	0.0055 (13)
C11	0.0390 (13)	0.0283 (13)	0.0341 (13)	0.0044 (11)	0.0094 (10)	0.0046 (11)
C12	0.0239 (10)	0.0255 (12)	0.0269 (11)	0.0055 (9)	−0.0005 (8)	−0.0051 (10)
C13	0.0413 (14)	0.0298 (14)	0.0296 (12)	−0.0048 (11)	−0.0048 (10)	−0.0012 (11)
C14	0.0528 (17)	0.0505 (18)	0.0244 (13)	0.0027 (15)	−0.0041 (11)	0.0001 (13)
C15	0.0420 (15)	0.072 (2)	0.0303 (13)	0.0034 (16)	−0.0085 (11)	−0.0144 (16)
C16	0.0305 (13)	0.066 (2)	0.0468 (16)	−0.0103 (15)	−0.0029 (11)	−0.0195 (17)
C17	0.0269 (12)	0.0416 (16)	0.0422 (14)	−0.0072 (12)	0.0034 (10)	−0.0072 (13)
C18	0.0321 (13)	0.0315 (14)	0.0318 (13)	−0.0024 (11)	−0.0098 (10)	0.0067 (11)
O19	0.0394 (11)	0.0384 (12)	0.0638 (14)	−0.0101 (10)	−0.0106 (10)	0.0201 (11)
O20	0.0399 (10)	0.0248 (9)	0.0376 (10)	0.0003 (8)	−0.0035 (8)	−0.0012 (8)
C21	0.0357 (12)	0.0308 (13)	0.0223 (10)	0.0026 (11)	0.0027 (9)	−0.0005 (10)
C22	0.0373 (13)	0.0370 (14)	0.0267 (12)	0.0071 (12)	0.0052 (10)	−0.0038 (11)
O23	0.0300 (8)	0.0340 (10)	0.0264 (8)	0.0074 (8)	0.0021 (6)	−0.0025 (8)
C24	0.0224 (10)	0.0262 (12)	0.0282 (11)	0.0001 (10)	0.0032 (8)	−0.0014 (10)
C25	0.0245 (11)	0.0280 (12)	0.0303 (12)	−0.0073 (10)	−0.0004 (9)	−0.0015 (10)
C26	0.0289 (12)	0.0327 (14)	0.0336 (13)	−0.0030 (11)	0.0009 (9)	−0.0074 (11)
C27	0.0428 (15)	0.0469 (18)	0.0350 (14)	−0.0127 (14)	−0.0017 (11)	−0.0100 (14)
C28	0.0585 (19)	0.0486 (18)	0.0344 (14)	−0.0137 (16)	−0.0135 (13)	−0.0068 (14)
C29	0.0542 (18)	0.0407 (18)	0.0456 (17)	0.0004 (15)	−0.0219 (14)	−0.0023 (14)
C30	0.0372 (13)	0.0318 (14)	0.0373 (14)	0.0023 (11)	−0.0080 (11)	−0.0043 (12)
O101	0.0258 (8)	0.0354 (10)	0.0223 (8)	−0.0028 (7)	−0.0020 (6)	0.0045 (7)
C102	0.0243 (11)	0.0282 (12)	0.0268 (11)	0.0022 (10)	−0.0011 (8)	0.0006 (10)
C103	0.0230 (10)	0.0315 (13)	0.0249 (11)	0.0045 (10)	0.0002 (8)	0.0039 (10)
O104	0.0251 (8)	0.0349 (10)	0.0219 (8)	0.0045 (7)	−0.0009 (6)	0.0021 (7)
C105	0.0276 (11)	0.0253 (12)	0.0234 (10)	0.0012 (10)	0.0017 (8)	0.0004 (10)
C106	0.0224 (11)	0.0293 (13)	0.0309 (12)	0.0040 (10)	0.0017 (9)	0.0040 (10)
C107	0.0379 (14)	0.0348 (15)	0.0367 (14)	−0.0038 (12)	−0.0050 (11)	0.0054 (12)
C108	0.0436 (16)	0.0373 (17)	0.0591 (19)	−0.0105 (14)	−0.0108 (13)	0.0091 (15)
C109	0.0315 (14)	0.0453 (19)	0.075 (2)	0.0004 (13)	0.0064 (14)	0.0245 (18)
C110	0.0442 (17)	0.052 (2)	0.060 (2)	0.0125 (16)	0.0272 (15)	0.0164 (17)
C111	0.0377 (14)	0.0385 (16)	0.0435 (15)	0.0096 (13)	0.0152 (12)	0.0036 (13)
C112	0.0268 (11)	0.0262 (12)	0.0230 (10)	0.0003 (10)	0.0003 (8)	−0.0033 (9)
C113	0.0333 (12)	0.0267 (13)	0.0356 (13)	−0.0018 (10)	−0.0012 (10)	0.0016 (11)
C114	0.0342 (14)	0.0329 (15)	0.0473 (15)	−0.0060 (12)	−0.0002 (11)	−0.0029 (13)
C115	0.0284 (12)	0.0387 (15)	0.0363 (13)	0.0009 (11)	−0.0051 (10)	−0.0111 (12)
C116	0.0329 (13)	0.0377 (15)	0.0239 (11)	0.0081 (11)	−0.0051 (9)	−0.0038 (11)
C117	0.0307 (12)	0.0303 (13)	0.0215 (10)	0.0026 (10)	0.0008 (9)	−0.0010 (10)
C118	0.0294 (12)	0.0332 (14)	0.0291 (12)	0.0039 (10)	−0.0005 (9)	0.0015 (11)
O119	0.0597 (13)	0.0362 (11)	0.0382 (10)	0.0110 (10)	0.0052 (9)	−0.0041 (10)
O120	0.0381 (10)	0.0317 (10)	0.0304 (9)	−0.0055 (8)	0.0030 (7)	0.0035 (8)
C121	0.0255 (11)	0.0375 (14)	0.0268 (11)	−0.0026 (11)	0.0028 (9)	−0.0006 (11)
C122	0.0268 (12)	0.0527 (18)	0.0278 (12)	−0.0056 (12)	0.0041 (9)	0.0032 (13)
O123	0.0274 (8)	0.0407 (11)	0.0264 (8)	−0.0048 (8)	0.0019 (6)	0.0068 (8)
C124	0.0308 (11)	0.0300 (13)	0.0231 (11)	−0.0002 (10)	0.0019 (9)	0.0024 (10)
C125	0.0297 (12)	0.0313 (13)	0.0247 (11)	−0.0025 (10)	0.0010 (9)	0.0040 (10)
C126	0.0334 (13)	0.0430 (16)	0.0294 (12)	0.0014 (12)	−0.0004 (10)	−0.0060 (12)
C127	0.0342 (13)	0.0577 (19)	0.0359 (14)	0.0035 (14)	0.0024 (11)	0.0010 (14)
C128	0.0325 (13)	0.0510 (19)	0.0407 (14)	−0.0064 (13)	−0.0058 (11)	0.0092 (14)
C129	0.0456 (16)	0.0385 (16)	0.0380 (14)	−0.0092 (13)	−0.0091 (12)	−0.0012 (13)
C130	0.0420 (14)	0.0284 (13)	0.0319 (13)	−0.0018 (12)	−0.0050 (11)	−0.0005 (11)

### Geometric parameters (Å, °)

**Table:**

O1—C2	1.432 (3)	O101—C102	1.429 (3)
O1—C24	1.422 (3)	O101—C124	1.425 (3)
C2—C3	1.518 (3)	C102—C103	1.511 (3)
C2—C21	1.527 (4)	C102—C121	1.526 (4)
C2—H21	1.000	C102—H1021	1.002
C3—O4	1.416 (3)	C103—O104	1.428 (3)
C3—C18	1.526 (4)	C103—C118	1.531 (4)
C3—H31	0.973	C103—H1031	0.991
O4—C5	1.451 (3)	O104—C105	1.448 (3)
C5—C6	1.518 (3)	C105—C106	1.516 (3)
C5—C12	1.512 (3)	C105—C112	1.521 (3)
C5—H51	1.009	C105—H1051	1.011
C6—C7	1.387 (4)	C106—C107	1.388 (4)
C6—C11	1.396 (3)	C106—C111	1.393 (4)
C7—C8	1.398 (4)	C107—C108	1.385 (4)
C7—H71	0.980	C107—H1071	0.938
C8—C9	1.374 (4)	C108—C109	1.377 (5)
C8—H81	0.952	C108—H1081	0.963
C9—C10	1.377 (5)	C109—C110	1.387 (5)
C9—H91	0.933	C109—H1091	0.930
C10—C11	1.390 (4)	C110—C111	1.388 (5)
C10—H101	0.969	C110—H1101	0.932
C11—H111	0.947	C111—H1111	0.941
C12—C13	1.388 (4)	C112—C113	1.393 (4)
C12—C17	1.382 (4)	C112—C117	1.393 (3)
C13—C14	1.389 (4)	C113—C114	1.395 (4)
C13—H131	0.939	C113—H1131	0.991
C14—C15	1.378 (5)	C114—C115	1.381 (4)
C14—H141	0.948	C114—H1141	0.964
C15—C16	1.374 (5)	C115—C116	1.380 (4)
C15—H151	0.946	C115—H1151	0.946
C16—C17	1.390 (4)	C116—C117	1.396 (3)
C16—H161	0.961	C116—H1161	0.960
C17—H171	0.916	C117—H1171	0.986
C18—O19	1.202 (3)	C118—O119	1.201 (3)
C18—O20	1.357 (3)	C118—O120	1.346 (3)
O20—C21	1.458 (3)	O120—C121	1.456 (3)
C21—C22	1.504 (4)	C121—C122	1.511 (3)
C21—H211	1.026	C121—H1211	0.994
C22—O23	1.428 (3)	C122—O123	1.430 (3)
C22—H221	1.021	C122—H1221	0.998
C22—H222	0.989	C122—H1222	0.998
O23—C24	1.402 (3)	O123—C124	1.409 (3)
C24—C25	1.511 (3)	C124—C125	1.503 (3)
C24—H241	0.997	C124—H1241	0.984
C25—C26	1.392 (4)	C125—C126	1.382 (4)
C25—C30	1.385 (4)	C125—C130	1.389 (4)
C26—C27	1.392 (4)	C126—C127	1.387 (4)
C26—H261	0.961	C126—H1261	0.953
C27—C28	1.385 (5)	C127—C128	1.386 (5)
C27—H271	0.961	C127—H1271	0.962
C28—C29	1.380 (5)	C128—C129	1.379 (5)
C28—H281	0.962	C128—H1281	0.955
C29—C30	1.394 (4)	C129—C130	1.394 (4)
C29—H291	0.986	C129—H1291	0.954
C30—H301	0.948	C130—H1301	0.977
			
C2—O1—C24	112.39 (17)	C102—O101—C124	112.03 (17)
O1—C2—C3	104.75 (18)	O101—C102—C103	106.20 (18)
O1—C2—C21	110.80 (19)	O101—C102—C121	111.3 (2)
C3—C2—C21	102.2 (2)	C103—C102—C121	102.0 (2)
O1—C2—H21	112.6	O101—C102—H1021	110.8
C3—C2—H21	112.2	C103—C102—H1021	117.7
C21—C2—H21	113.5	C121—C102—H1021	108.5
C2—C3—O4	108.98 (18)	C102—C103—O104	107.53 (19)
C2—C3—C18	102.1 (2)	C102—C103—C118	102.1 (2)
O4—C3—C18	106.5 (2)	O104—C103—C118	107.4 (2)
C2—C3—H31	110.6	C102—C103—H1031	115.0
O4—C3—H31	113.3	O104—C103—H1031	112.1
C18—C3—H31	114.7	C118—C103—H1031	112.1
C3—O4—C5	112.91 (17)	C103—O104—C105	113.76 (17)
O4—C5—C6	110.32 (19)	O104—C105—C106	111.0 (2)
O4—C5—C12	105.39 (18)	O104—C105—C112	105.51 (17)
C6—C5—C12	114.4 (2)	C106—C105—C112	114.5 (2)
O4—C5—H51	107.6	O104—C105—H1051	106.0
C6—C5—H51	107.6	C106—C105—H1051	110.5
C12—C5—H51	111.3	C112—C105—H1051	108.9
C5—C6—C7	121.7 (2)	C105—C106—C107	122.0 (2)
C5—C6—C11	119.1 (2)	C105—C106—C111	118.6 (2)
C7—C6—C11	119.2 (2)	C107—C106—C111	119.4 (3)
C6—C7—C8	120.3 (3)	C106—C107—C108	120.6 (3)
C6—C7—H71	120.5	C106—C107—H1071	119.8
C8—C7—H71	119.2	C108—C107—H1071	119.6
C7—C8—C9	119.7 (3)	C107—C108—C109	119.9 (3)
C7—C8—H81	119.1	C107—C108—H1081	121.1
C9—C8—H81	121.2	C109—C108—H1081	119.0
C8—C9—C10	120.6 (3)	C108—C109—C110	120.1 (3)
C8—C9—H91	119.5	C108—C109—H1091	121.1
C10—C9—H91	119.9	C110—C109—H1091	118.7
C9—C10—C11	120.1 (3)	C109—C110—C111	120.2 (3)
C9—C10—H101	119.7	C109—C110—H1101	120.7
C11—C10—H101	120.1	C111—C110—H1101	119.1
C6—C11—C10	120.0 (3)	C106—C111—C110	119.8 (3)
C6—C11—H111	120.7	C106—C111—H1111	119.4
C10—C11—H111	119.3	C110—C111—H1111	120.8
C5—C12—C13	119.8 (2)	C105—C112—C113	118.8 (2)
C5—C12—C17	120.8 (2)	C105—C112—C117	122.6 (2)
C13—C12—C17	119.4 (2)	C113—C112—C117	118.6 (2)
C12—C13—C14	120.5 (3)	C112—C113—C114	120.9 (3)
C12—C13—H131	122.2	C112—C113—H1131	119.8
C14—C13—H131	117.3	C114—C113—H1131	119.2
C13—C14—C15	119.6 (3)	C113—C114—C115	119.7 (3)
C13—C14—H141	119.9	C113—C114—H1141	119.0
C15—C14—H141	120.5	C115—C114—H1141	121.2
C14—C15—C16	120.3 (3)	C114—C115—C116	120.1 (2)
C14—C15—H151	122.4	C114—C115—H1151	120.3
C16—C15—H151	117.4	C116—C115—H1151	119.6
C15—C16—C17	120.4 (3)	C115—C116—C117	120.2 (2)
C15—C16—H161	122.9	C115—C116—H1161	118.9
C17—C16—H161	116.7	C117—C116—H1161	120.8
C16—C17—C12	119.9 (3)	C116—C117—C112	120.3 (2)
C16—C17—H171	120.0	C116—C117—H1171	122.7
C12—C17—H171	120.0	C112—C117—H1171	116.9
C3—C18—O19	128.2 (3)	C103—C118—O119	128.2 (3)
C3—C18—O20	109.4 (2)	C103—C118—O120	109.7 (2)
O19—C18—O20	122.3 (3)	O119—C118—O120	122.0 (3)
C18—O20—C21	110.2 (2)	C118—O120—C121	110.0 (2)
C2—C21—O20	104.14 (18)	C102—C121—O120	104.57 (19)
C2—C21—C22	113.2 (2)	C102—C121—C122	113.7 (2)
O20—C21—C22	110.3 (2)	O120—C121—C122	109.8 (2)
C2—C21—H211	110.3	C102—C121—H1211	109.4
O20—C21—H211	109.0	O120—C121—H1211	107.1
C22—C21—H211	109.7	C122—C121—H1211	111.9
C21—C22—O23	111.80 (19)	C121—C122—O123	111.63 (19)
C21—C22—H221	108.5	C121—C122—H1221	108.8
O23—C22—H221	112.7	O123—C122—H1221	109.1
C21—C22—H222	109.5	C121—C122—H1222	108.2
O23—C22—H222	105.2	O123—C122—H1222	110.5
H221—C22—H222	109.0	H1221—C122—H1222	108.5
C22—O23—C24	110.9 (2)	C122—O123—C124	110.2 (2)
O1—C24—O23	110.67 (19)	O101—C124—O123	109.8 (2)
O1—C24—C25	107.49 (18)	O101—C124—C125	108.28 (18)
O23—C24—C25	108.6 (2)	O123—C124—C125	109.0 (2)
O1—C24—H241	109.8	O101—C124—H1241	109.1
O23—C24—H241	109.3	O123—C124—H1241	110.5
C25—C24—H241	111.0	C125—C124—H1241	110.2
C24—C25—C26	119.5 (2)	C124—C125—C126	120.9 (2)
C24—C25—C30	120.7 (2)	C124—C125—C130	119.3 (2)
C26—C25—C30	119.9 (2)	C126—C125—C130	119.8 (2)
C25—C26—C27	120.0 (3)	C125—C126—C127	120.2 (3)
C25—C26—H261	122.5	C125—C126—H1261	119.7
C27—C26—H261	117.5	C127—C126—H1261	120.0
C26—C27—C28	120.0 (3)	C126—C127—C128	120.0 (3)
C26—C27—H271	120.1	C126—C127—H1271	119.9
C28—C27—H271	119.9	C128—C127—H1271	120.0
C27—C28—C29	120.0 (3)	C127—C128—C129	120.1 (3)
C27—C28—H281	119.3	C127—C128—H1281	119.0
C29—C28—H281	120.7	C129—C128—H1281	121.0
C28—C29—C30	120.3 (3)	C128—C129—C130	120.0 (3)
C28—C29—H291	120.5	C128—C129—H1291	119.4
C30—C29—H291	119.2	C130—C129—H1291	120.7
C29—C30—C25	119.8 (3)	C129—C130—C125	119.9 (3)
C29—C30—H301	118.6	C129—C130—H1301	120.3
C25—C30—H301	121.6	C125—C130—H1301	119.8

### Hydrogen-bond geometry (Å, °)

**Table:**

*D*—H···*A*	*D*—H	H···*A*	*D*···*A*	*D*—H···*A*
C8—H81···O19^i^	0.95	2.57	3.520 (4)	173
C14—H141···O119^ii^	0.95	2.55	3.309 (4)	137
C28—H281···O19^iii^	0.96	2.57	3.225 (4)	126

Symmetry codes: (i) *x*, *y*+1, *z*; (ii) *x*, *y*, *z*−1; (iii) −*x*+1, *y*+1/2, −*z*+1.
